# Molecular changes during extended neoadjuvant letrozole treatment of breast cancer: distinguishing acquired resistance from dormant tumours

**DOI:** 10.1186/s13058-018-1089-5

**Published:** 2019-01-07

**Authors:** Cigdem Selli, Arran K. Turnbull, Dominic A. Pearce, Ang Li, Anu Fernando, Jimi Wills, Lorna Renshaw, Jeremy S. Thomas, J. Michael Dixon, Andrew H. Sims

**Affiliations:** 1 Applied Bioinformatics of Cancer, University of Edinburgh Cancer Research UK Centre, MRC Institute of Genetics and Molecular Medicine, Edinburgh, UK; 2 Department of Pharmacology, Faculty of Pharmacy, Ege University, 35040 Izmir, Turkey; 3 Edinburgh Breast Unit, Western General Hospital, Edinburgh, UK; 4 Mass Spectrometry Unit, MRC Institute of Genetics and Molecular Medicine, Edinburgh, UK

**Keywords:** Dormancy, Oestrogen deprivation therapy, Epigenetics, Letrozole, Sequential samples, Resistance, Microarray, Proteomics

## Abstract

**Background:**

The risk of recurrence for endocrine-treated breast cancer patients persists for many years or even decades following surgery and apparently successful adjuvant therapy. This period of dormancy and acquired resistance is inherently difficult to investigate; previous efforts have been limited to in-vitro or in-vivo approaches. In this study, sequential tumour samples from patients receiving extended neoadjuvant aromatase inhibitor therapy were characterised as a novel clinical model.

**Methods:**

Consecutive tumour samples from 62 patients undergoing extended (4–45 months) neoadjuvant aromatase inhibitor therapy with letrozole were subjected to transcriptomic and proteomic analysis, representing before (≤ 0), early (13–120 days), and long-term (> 120 days) neoadjuvant aromatase inhibitor therapy with letrozole. Patients with at least a 40% initial reduction in tumour size by 4 months of treatment were included. Of these, 42 patients with no subsequent progression were classified as “dormant”, and the remaining 20 patients as “acquired resistant”.

**Results:**

Changes in gene expression in dormant tumours begin early and become more pronounced at later time points. Therapy-induced changes in resistant tumours were common features of treatment, rather than being specific to the resistant phenotype. Comparative analysis of long-term treated dormant and resistant tumours highlighted changes in epigenetics pathways including DNA methylation and histone acetylation. The DNA methylation marks 5-methylcytosine and 5-hydroxymethylcytosine were significantly reduced in resistant tumours compared with dormant tissues after extended letrozole treatment.

**Conclusions:**

This is the first patient-matched gene expression study investigating long-term aromatase inhibitor-induced dormancy and acquired resistance in breast cancer. Dormant tumours continue to change during treatment whereas acquired resistant tumours more closely resemble their diagnostic samples. Global loss of DNA methylation was observed in resistant tumours under extended treatment. Epigenetic alterations may lead to escape from dormancy and drive acquired resistance in a subset of patients, supporting a potential role for therapy targeted at these epigenetic alterations in the management of resistance to oestrogen deprivation therapy.

**Electronic supplementary material:**

The online version of this article (10.1186/s13058-018-1089-5) contains supplementary material, which is available to authorized users.

## Background

Approximately 70% of breast cancer patients who have oestrogen receptor (ER) alpha-positive tumours receive adjuvant oestrogen deprivation therapy. Five years of aromatase inhibitor therapy produces a 40% reduction in 10-year mortality [1]. However, while the annual risk of mortality for ER-negative breast cancer decreases following the first 5 years after diagnosis, the annual rate remains constant for ER^+^ patients [2]. In fact, women with ER^+^ early-stage disease treated with 5 years of adjuvant endocrine therapy have a persistent risk of recurrence and death from breast cancer for at least 20 years after diagnosis [3]. Molecular studies have demonstrated that nodal and distant metastases are highly similar to their matched primary tumours, implicating a continuation of the original cancer [4–6]. However, the time between treatment and recurrence is often greater than that which can be explained by normal cell-doubling rates [7], implying cancer cells remain dormant in the body before re-awakening.

Residual dormant cancer cells are hypothesised to persist either by withdrawing from the cell cycle and transitioning to a quiescence state or by continuing to proliferate at a reduced rate, counter-balanced by cell death [8]. Reawakened dormant cells may become detectable after reaching a detection threshold or reactivated via increased angiogenesis, and/or escape from the inhibitory microenvironment or immune effects [9, 10]. Dormancy is therefore considered a major mechanism underlying resistance to therapy, where dormant cells survive despite anti-proliferative oestrogen deprivation therapy.

Resistance to oestrogen deprivation therapy may occur at disease inception (de novo or innate resistance), but a larger proportion of patients acquire resistance during treatment (acquired/secondary resistance) [11]. Several mechanisms of resistance to oestrogen deprivation therapy have been described previously [12, 13]. However, the majority of these findings are based on preclinical data obtained from cell lines and animal models. It is therefore difficult to know if these accurately reflect molecular changes in patient tumours.

Expression profiling of clinical samples, measuring the effect of, or predicting response to, treatment has recently become feasible. However, experimental design issues, such as the difficulty in obtaining paired samples for comparison, particularly for longer time intervals, makes it difficult to study changes within tumours [14]. For example, a previous study investigating tamoxifen failure compared samples from patients requiring salvage surgery with pre-treatment samples from an unrelated group of disease-free patients [15]. More recently, sequential patient-matched samples have been successfully utilised to determine treatment-induced dynamic changes in tumours at 2 weeks to 3 months, demonstrating the effectiveness of this approach [16–18].

For a variety of reasons, including being unfit for surgery, a proportion of patients receiving pre-surgical oestrogen deprivation therapy do not have their tumours excised following 3–4 months of treatment. These long-term endocrine-treated tumours represent a unique group that can inform how tumours respond to extended oestrogen deprivation in situ. Having initially shrunk in size, some tumours remain at a steady volume and appear dormant, whilst others subsequently begin to regrow. We have utilised this unique cohort of sequential samples from patients receiving extended neoadjuvant oestrogen deprivation therapy to characterise luminal breast cancer dormancy and acquired resistance as a novel clinical model.

## Methods

### Patients and samples

**Fig. 1:**
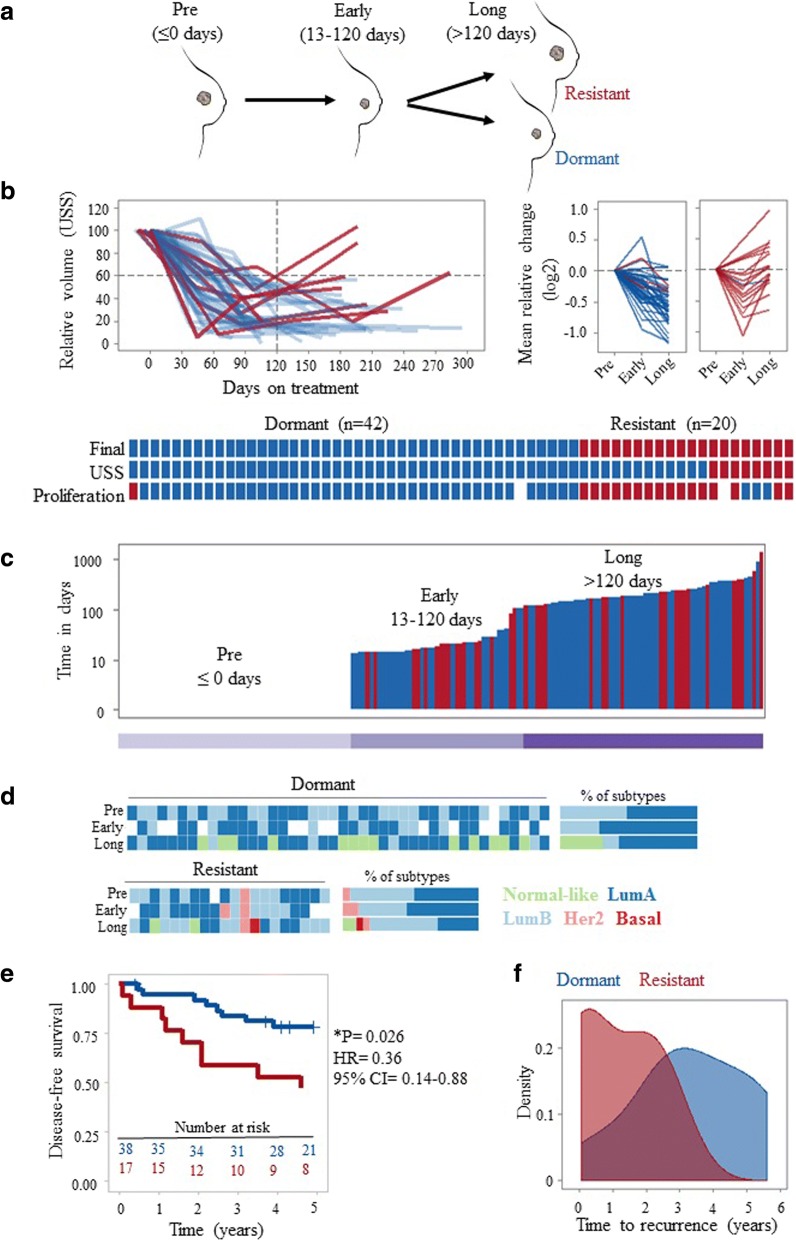
Long-term oestrogen deprivation therapy as a clinical model to investigate breast cancer dormancy and acquired resistance. **a** Extended (4–45 months) letrozole treatment was exploited as a clinical model of breast cancer dormancy and acquired resistance. Sequential clinical samples from the same patient with no surgery and extended treatment were used to model clinical breast cancer dormancy and resistance. Before (pre, ≤ 0 days), early-on (early, 13–120 days) and long-term (long, > 120 days) neoadjuvant aromatase inhibitor therapy with letrozole. **b** Dynamic change in tumour size by ultrasound scan (USS) and mean expression of proliferation markers MKI67, PCNA, and MCM2 were used to classify patients into two categories: dormant (blue) and resistant (red). Overall comparisons of classifications per patient based on USS and mean change in proliferation markers with final classification are shown. **c** The duration of letrozole treatment (days) for samples from dormant (blue) and resistant (red) patients. Each bar represents a sample. Samples are ordered by time on treatment. **d** Intrinsic subtype classification by PAM50 of samples at each time point. Stacked bar graphs on the right show the percentage of each subtype of samples from dormant and resistant patients. **e** Kaplan-Meier plot showing disease-free survival probability in patients with dormant versus resistant tumours (log-rank test). Disease-free survival was defined from time of surgery. **f** Density plot showing the distribution of time to recurrence (in years; defined from time of surgery) in patients with dormant and resistant tumours. CI confidence interval, HR hazard ratio, LumA luminal A, LumB luminal B

Breast cancer patients were treated with neoadjuvant aromatase inhibitor therapy with letrozole (Femara, 2.5 mg; Novartis Pharma AG, Basel, Switzerland) for a minimum of 4 months; tumours were not removed either because patients declined or were unfit for surgery. The study was approved by the local regional ethics committee (07/S1103/26, August 2007) and all patients gave informed consent. Clinical characteristics of the tumours are given in Table 1. A consort diagram detailing inclusion and exclusion criteria is provided in Additional file 1 (Figure S1). Patients with > 40% initial decrease in tumour size by 4 months of treatment were included in the study. Those with no subsequent progression on imaging by the latest biopsy were classified as “dormant”; otherwise, they were classified as “acquired resistant” (Fig. 1a, b). For patients whose latest ultrasound scan (USS) measurement was taken more than a month before surgery, changes in gene expression (mean relative change) of three widely used proliferation markers (MKI67, PCNA, and MCM2) were used to assist classification. Tumours with an increase in proliferation marker expression (either after an initial decrease or not) were classified as “acquired resistant”, otherwise there were classified as “dormant”. Sequential tumour biopsies were taken with a 14-gauge needle before and after letrozole treatment and at the time of surgery. Fresh samples were snap-frozen in liquid nitrogen and each tumour sample was confirmed to contain ≥ 50% cellularity and at least 60% tumour tissue using haematoxylin and eosin (H&E) sections. Following pulverisation of tissue with a membrane disruptor (Micro-Dismembrator U, Braun Biotech), phase separation was performed by guanidinium thiocyanate-phenol-chloroform extraction (Qiazol Lysis Reagent).

**Table 1:** Patient characteristics

	Dormant, *n* (%)	Resistant, *n* (%)	Total, *n*	*p* value^a^
Total no. of patients	42	20	62	
Total no. of samples	111	56	167	
Age at diagnosis				
Mean	75	72		
Range	53–87	56–89		
Tumour grade				0.39
1	6 (14.3)	1 (5.0)	7	
2	27 (64.3)	10 (50.0)	37	
3	8 (19.0)	6 (30.0)	14	
NA	1 (2.4)	3 (15.0)	4	
Tumour size				0.71
T1	5 (11.9)	4 (20.0)	9	
T2	19 (45.2)	9 (45.0)	28	
T3	2 (4.8)	2 (10.0)	4	
T4	11 (26.2)	4 (20.0)	15	
NA	5 (11.9)	1 (5.0)	6	
Nodal status				0.36
N0	27 (64.3)	11 (55.0)	38	
N1	8 (19.0)	7 (35.0)	15	
N2	1 (2.4)	1 (5.0)	2	
N3	1 (2.4)	0	1	
NX	1 (2.4)	0	1	
NA	4 (9.5)	1 (5.0)	5	
Metastasis status				1.00
M0	34 (80.9)	18 (90.0)	56	
M1	2 (4.8)	0	2	
MX	1 (2.4)	1 (5.0)	2	
NA	5 (11.9)	1 (5.0)	6	
ER score (Allred)				0.18
6	1 (2.4)	0	1	
7	6 (14.3)	6 (30.0)	12	
8	35 (83.3)	14 (70.0)	49	
HER status				0.69
Negative	35 (83.3)	12 (60.0)	47	
Positive	6 (14.3)	3 (15.0)	9	
NA	1 (2.4)	5 (25.0)	6	
Histological type				0.73
IDC (no special type)	18 (42.9)	6 (30)	24	
ILC	8 (19.0)	4 (20)	12	
Mucinous	1 (2.4)	0	1	
NA	15 (35.7)	10 (50)	25	
Molecular subtype^b^				1.00
Luminal A	21 (50.0)	9 (45.0)	30	
Luminal B	20 (47.6)	9 (45.0)	29	
HER2 enriched	0	1 (5.0)	1	
Basal-like	0	0	0	
Normal-like	0	0	0	
NA	1 (2.4)	1 (5.0)	2	

*ER* oestrogen receptor, *IDC* invasive ductal carcinoma, *ILC* invasive lobular carcinoma, *NA* not available

^a^Fisher exact test (*p* < 0.05, two-tailed)

^b^At diagnosis by PAM50 (genefu)

### Gene expression profiling and analysis

RNA was extracted from the aqueous phase by column-based purification (RNeasy mini kit, Qiagen) and then labelled and hybridized (HumanHT-12 v4 Illumina BeadChip) according to the manufacturer’s protocol (NuGEN) as previously described [19, 20]. Raw data were detection (*p* < 0.05, ≥ 3 samples) and quality filtered, log_2_ transformed, and quantile normalized using the Bioconductor lumi package [21]. Data are publicly available from NCBI GEO under accession GSE111563. The analysis also includes data from 14 patients (42 samples, GSE59515) and 9 patients (24 samples, GSE55374) from previous studies [16, 19] which meet the criteria defined above; the relationship between the samples from these datasets is indicated in Additional file 2 (Table S1). Hierarchical clustering analysis was performed using a complete linkage method and Euclidean distance. Pathway enrichment analysis and visualisation were performed using ReactomePA [22]. Differential gene expression analysis was performed with Rank Products [23]. The significance of differences was evaluated by using unpaired Wilcoxon test for two groups and analysis of variance (ANOVA) with post-hoc Tukey HSD for multiple comparisons.

### Proteomics analysis

Proteins were isolated from the organic phase of Qiazol [24]. Pellets were sonicated and dissolved in 1% SDS. Proteomics was performed using Thermo Q Exactive plus and Label-free Quantitation (LFQ). Peptides obtained from samples were analysed in mass spectrometry runs; serial samples from the patients were run on the same day. A modified version of Filter Aided Sample Preparation (FASP) was performed using serial digests with lysC and trypsin to generate two orthogonal fractions per sample [25, 26]. The mass spectrometry spectra generated in each run were used for relative quantitation of individual peptides. Normalization and quantifications of peptides were performed using MaxLFQ and MaxQuant [27]. A total of 6251 protein groups were identified. Data was log_2_ transformed and missing values were imputed as the minimum observed value in each sample. The data have been deposited to the ProteomeXchange Consortium via the PRIDE [28] partner repository with the dataset identifier PXD009328.

### Immunohistochemistry and scoring

Formalin-fixed paraffin-embedded (FFPE) sections were processed using an automated stainer (Leica Biosystems, Bond III). Heat-induced epitope retrieval for both antibodies was performed by 30-min incubation in citrate-based pH 6.0 epitope retrieval (ER1) solution followed by incubation in 3.5 N HCl for 15 min at room temperature as suggested by Haffner et al. [29]. For 5-methylcytosine (5-mC) and 5-hydroxymethylcytosine (5-hmC) detection, mouse monoclonal 5-methylcytosine specific (33D3; Abcam, ab10805) and rabbit polyclonal 5-hydroxylmethylcytosine (Active Motif, 39,769) antibodies were used, respectively. Both antibodies were used at 1/1000 dilution and were incubated for 15 min. Detection was performed using secondary antibody-horseradish peroxidase (HRP) conjugates and substrate-chromogen (3,3’-diaminobenzidine (DAB)). After staining, slides were counterstained with haematoxylin. Nuclear staining in epithelial cells was evaluated using an H-score obtained by multiplying the intensity of the stain (0: no staining; 1: weak staining; 2: moderate staining; 3: intense staining) by the percentage of cells (H-score range, 0 to 300).

## Results

### Long-term oestrogen deprivation therapy as a model of dormancy and acquired resistance

A cohort of 62 primary breast cancer patients receiving at least 4 months of oestrogen deprivation therapy (Fig. 1a) and initially responding were stratified into two groups, ‘dormant’ and ‘acquired resistant’ based on dynamic changes in tumour size and proliferation (see methods and Fig. 1b). Patient-matched sequential samples were available at three time points: before (≤ 0 days), early (13–120 days), and long-term (> 120 days) treatment. Dormant and acquired resistant samples were distributed uniformly with respect to time on treatment, and duration at each time point was not significantly different between response groups (Table 1). For long-term treatment, the mean and range were 186 (121–884) days and 226 (121–1366) days for patients with dormant and acquired resistant tumours, respectively (Fig. 1c).

There were no significant differences in patient clinico-pathological features between response classes before treatment (Table 1). However, prediction analysis of microarray (PAM)50 intrinsic molecular subtypes were found to change during oestrogen deprivation therapy (Fig. 1d). These changes were consistent with known associations with outcome, with all dormant tumours either remaining the same or switching to better prognosis luminal A or normal-like tumours. For resistant tumours, however, 25% (5 out of 20) switched to a subtype of worse prognosis (Fig. 1d). The proportion of luminal B tumours characterised by reduced endocrine sensitivity and higher proliferation was higher in resistant tumours compared with dormant tumours under early (35% versus 27%) and long-term (50% versus 12%) treatment (Fig. 1d; stacked bar graphs on the right). The PAM50 defines breast cancer into four intrinsic molecular subtypes: luminal A, luminal B, HER2-enriched, and basal-like [30]. PAM50 intrinsic subtyping has been shown to provide additional prognostic value to standard clinicopathological factors where luminal A tumours had a significantly better outcome than luminal B, HER2-enriched, and basal-like tumours [31].

As expected, Kaplan-Meier survival analysis demonstrated significantly worse outcomes for patients with resistant tumours compared with patients with dormant tumours (log rank, *p* = 0.026; Fig. 1e). Recurrence rates for patients with dormant and resistant tumours were 21% (9/42) and 45% (9/20), respectively. Moreover, patients with resistant tumours suffered significantly earlier recurrences compared with patients with dormant tumours (*p* = 0.05; range 26–947 versus 136–2042 days; Fig. 1f). Disease-free survival and time to recurrence were defined from time of surgery, not from the time of diagnosis, since patient classification was performed based on change in tumour size by USS and proliferation by gene expression at on-treatment and surgery time points.

### Distinct transcriptomic changes under long-term letrozole treatment

**Fig. 2:**
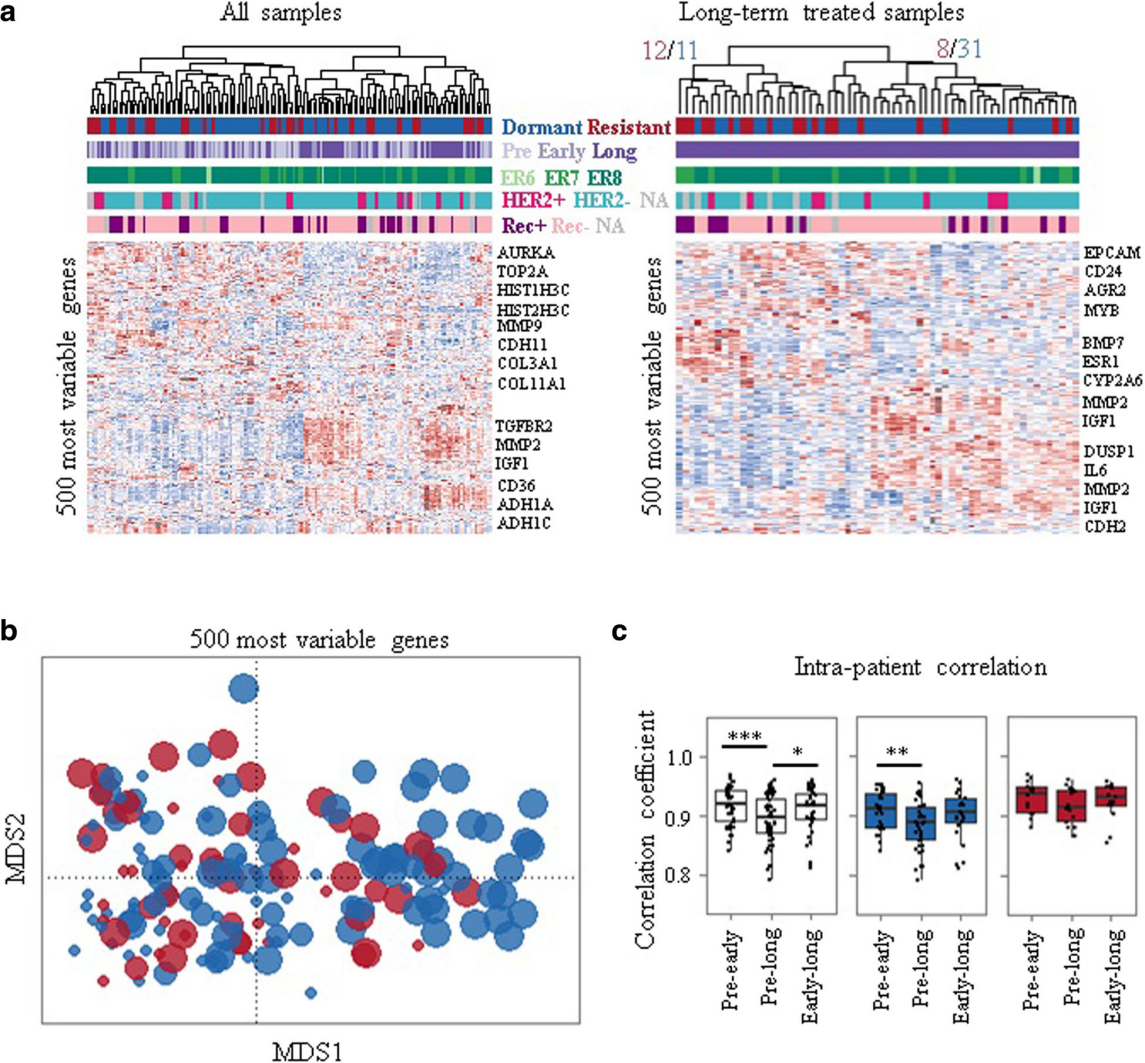
Distinct transcriptomic changes during long-term aromatase inhibitor treatment. **a** Unsupervised hierarchical clustering with most variant 500 genes across all samples and long-term treated samples. ER6, ER7, ER8 correspond to oestrogen receptor (ER) Allred scores 6, 7, and 8, respectively. **b** Multidimensional scaling (MDS) plot using the 500 genes with the highest variance across all time points. Each dot corresponds to a sample and sizes represent the duration of treatment. **c** Intra-patient (comparison of samples from the same patient) correlations of transcriptome are shown. Dormant (blue); resistant (red); before (pre, ≤ 0 days), early-on (early, 13–120 days), and long-term (long, > 120 days) neoadjuvant aromatase inhibitor therapy with letrozole. ***p < 0.001; **p < 0.01; *p < 0.05. NA not available, Rec+ recurrence, Rec– recurrence free

Unsupervised analysis was performed to consider whether sequential samples displayed greater similarity between response classes or treatment duration. Hierarchical clustering using the 500 genes with the highest variance across all samples revealed two main subclasses, seemingly driven by time on treatment, with resistant and dormant tumours indistinguishable (Fig. 2a). The dominant pattern was that the samples of the same patient usually clustered together (Fig. 2a).

When long-term treated samples were considered alone, two clusters did emerge, the larger of which contained mostly dormant samples (79%), whilst the second had a roughly even proportion of dormant (48%) and resistant (52%) samples (Fig. 2a). Similarly, a multidimensional scaling (MDS) plot for the 500 genes with the highest variance across all time points revealed consistent changes over time in response to treatment for both dormant and acquired resistant samples (Fig. 2b), although long-term dormant samples were much more distinguishable from pre-treatment samples than the long-term acquired resistant samples (Fig. 2b).

Correlations between tumours from different individuals (inter-patient) remained similar at each time point and were not different between response classes (data not shown) as corroborated by hierarchical clustering analysis with all samples and across all the time points. However, correlations of the transcriptome between matched sequential samples (intra-patient) revealed that pre-treatment samples were significantly (*p* = 0.01) less similar to their long-term treated pairs (median 0.89, range 0.74–0.95) than their early treatment pairs (median 0.91, range 0.84–0.95) (Fig. 2c). However, when divided by dormancy status this finding was only significant (*p* = 0.01) for dormant tumours (Fig. 2c), suggesting that dormant tumours continue to diverge transcriptionally whereas acquired resistant tumours do not consistently differ after initial or extended treatment, as mirrored in the MDS representation (Fig. 2b).

Perhaps surprisingly, oestrogen, progesterone, and androgen receptors and their target genes [32] were not differentially expressed between long-term treated dormant and resistant tumours (data not shown).

### Changes in gene expression/pathways following long-term letrozole treatment

**Fig. 3:**
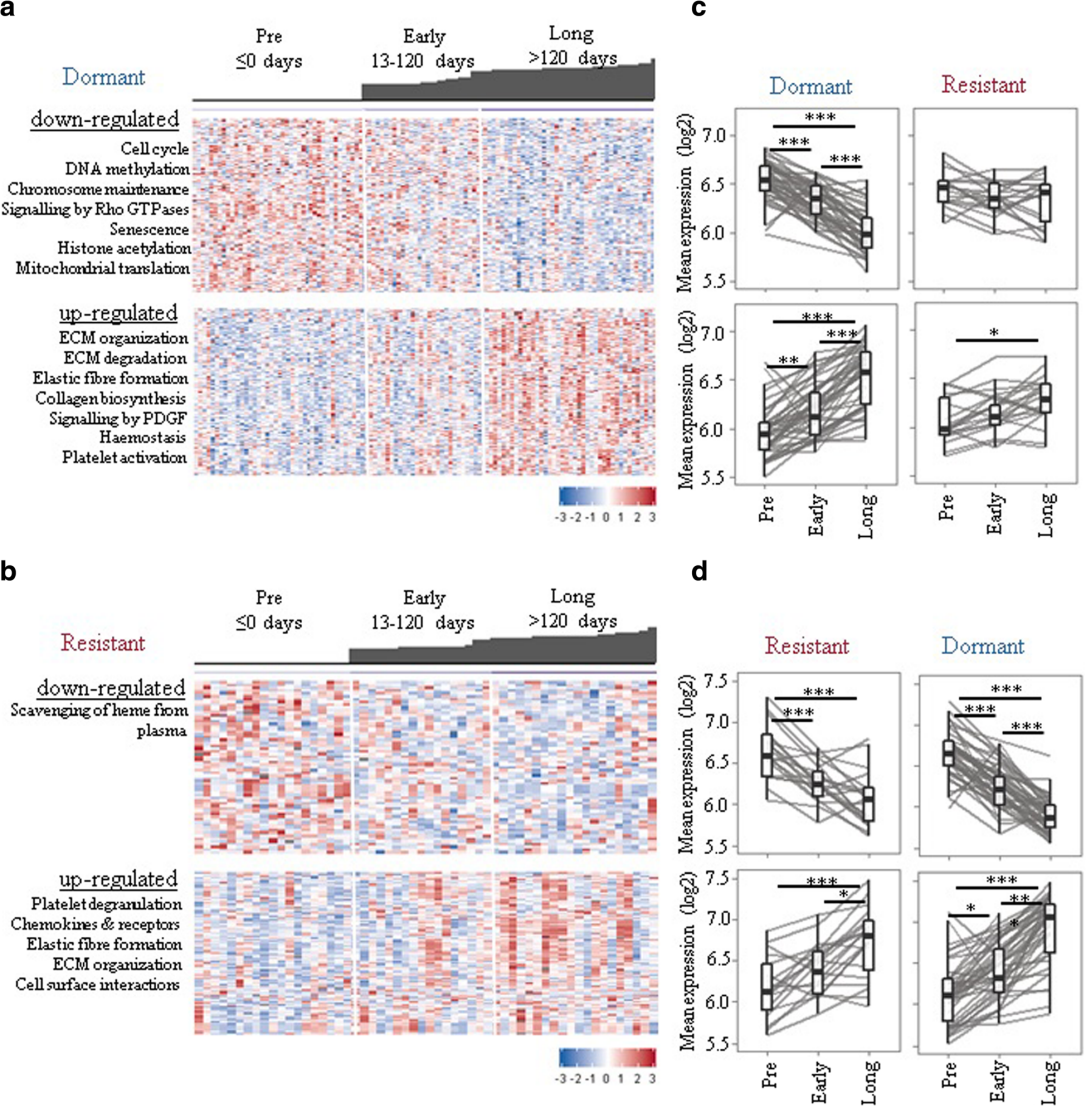
Long-term oestrogen deprivation therapy is associated with cell cycle, senescence, epigenetic regulation, and extracellular matrix (ECM)-associated pathways. Differentially expressed genes between pre-treatment and long-term treated dormant (**a**) and resistant (**b**) tumours were determined. Heat-maps showing change in downregulated and upregulated gene expression in dormant (**a**) and resistant (**b**) samples. Each column represents a sample and each row a gene. Colours are log_2_ mean-centred values with red indicating high values and blue indicating low expression. Bar plots on top of heat-maps represent the time on treatment (days) for each sample. **c**,**d** Graphs show dynamic changes in mean expression of differentially expressed genes in response classes. ***p < 0.001; **p < 0.01; *p < 0.05

To consider whether the gene expression changes due to treatment in the dormant and acquired resistant tumours were the same or distinct we initially considered them separately. Pairwise rank product analysis (pre- versus long-term treatment, false discovery rate (FDR) < 0.01) identified 2319 genes significantly differentially expressed (1063 downregulated and 1256 upregulated) between long-term treated and pre-treatment dormant tumours (Additional file 2: Table S2). These genes were significantly enriched (*p* < 0.01) for a total of 62 and 26 pathways, respectively (Additional file 2: Table S3), including reductions in cell cycle, senescence, DNA methylation, and an increase in extracellular matrix (ECM) organization. These findings are consistent with previous studies of patient-matched sequential samples treated with oestrogen deprivation therapy [16–18]. Acquired resistant tumours displayed much fewer consistently differentially expressed genes (238; 63 downregulated and 175 upregulated) between long-term treated and pre-treatment samples (Additional file 2: Table S4). Genes that were upregulated in resistant tumours (pre-treatment versus long-term treatment) were enriched for several of the same pathways as dormant tumours (ECM organization, elastic fibre formation, and platelet degranulation), but downregulated genes were much more variable (Additional file 2: Table S5; Fig. 3a, b).

Having determined that dormant and acquired resistant tumours have somewhat distinct changes during treatment at the molecular level, the question remained as to whether these changes tend to occur at earlier time points or were specific to long-term treatment. For dormant tumours, differential expression begins early on, but becomes more pronounced at later time points (Fig. 3a). Downregulated genes (pre-treatment versus long-term treatment) were most evident at early-on treatment for resistant tumours, consistent with their initial response to treatment, whilst upregulated genes (pre-treatment versus long-term treatment) were most changed after long-term treatment, potentially suggesting that these genes may mediate acquired resistance (Fig. 3b). We further examined whether differentially expressed genes between pre-treatment versus long-term treatment identified in each response class (dormant and resistant) were shared (Fig. 3c, d). Both downregulated and upregulated genes identified in resistant tumours were significantly changed (*p* < 0.01) in dormant tumours (Fig. 3d). However, only upregulated genes identified in dormant tumours were significantly upregulated in resistant tumours without any change in downregulated genes (Fig. 3c), implicating a partial lack of response to treatment at the molecular level in acquired resistance patients.

### A potential role of epigenetic regulation in acquired resistance

**Fig. 4:**
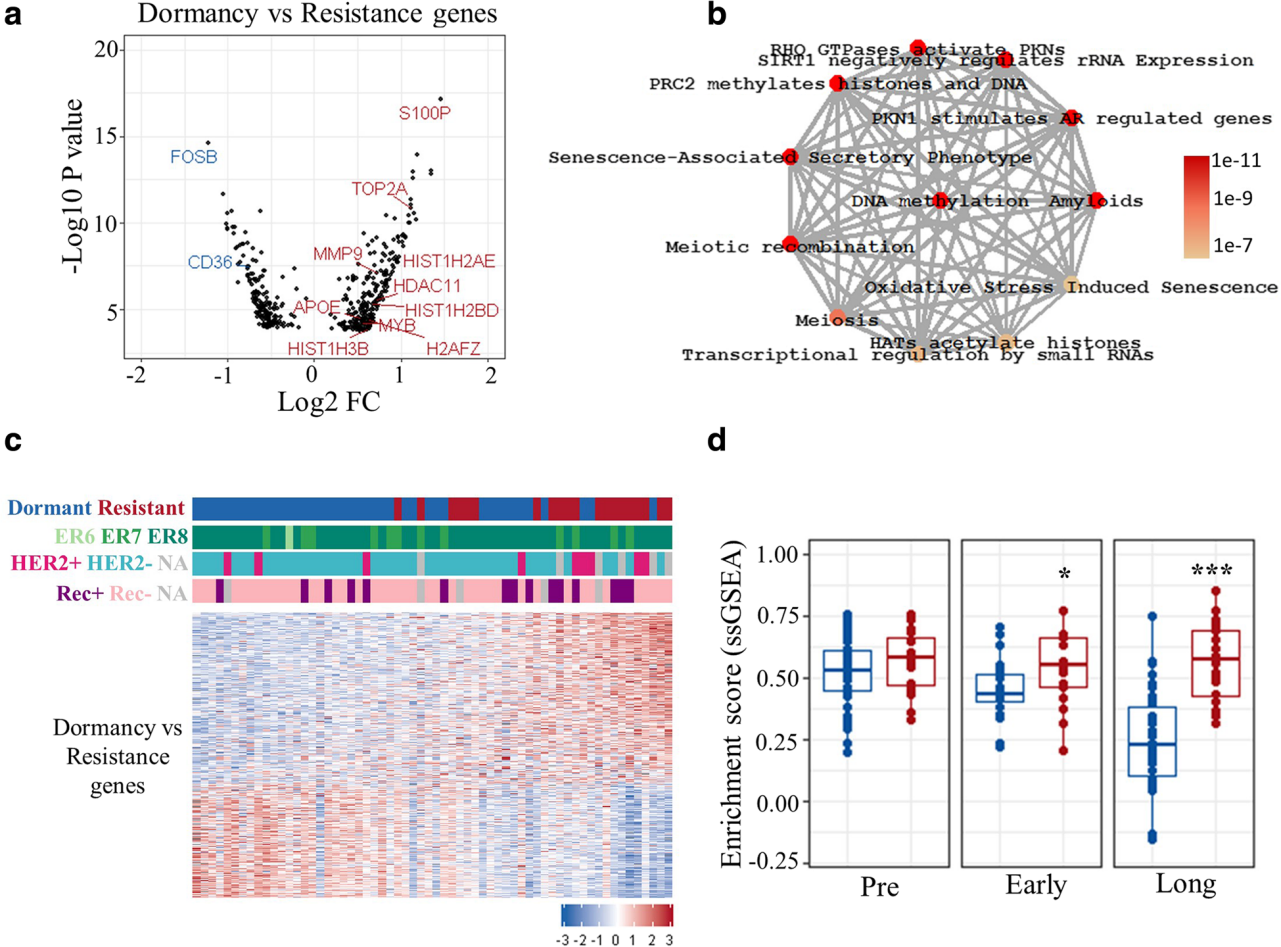
Comparative analysis of dormant and resistant tumours. **a** Volcano plot showing differentially expressed genes between long-term treated dormant and resistant tumours (dormancy versus resistance genes). Some upregulated and downregulated genes in resistant tumours are highlighted in red and blue, respectively. **b** Significantly enriched pathways for dormancy versus resistance genes (*p* < 0.01; ReactomePA). Grey edges connecting the nodes indicates genes shared between the nodes/pathways, and the width of the edge is scaled by the number of common genes. Colours indicates the significance (*p* value) where red is a lower *p* value. **c** Heatmap showing partial separation of long-term treated dormant and resistant samples using dormancy versus resistance genes (a total of 419; 170 downregulated and 249 upregulated genes). Colours are log_2_ mean-centred values with red indicating high and blue indicating low expression. Genes are sorted by fold-change (FC) values from most to least up/downregulated. Samples are sorted by sum expression of upregulated genes. ER6, ER7, and ER8 correspond to oestrogen receptor (ER) Allred scores 6, 7, and 8, respectively. **d** Comparison of single sample gene enrichment analysis (ssGSEA) scores of dormancy versus resistance upregulated genes between dormant and acquired resistant tumours. Dormant (blue); resistant (red); before (pre, ≤ 0 days), early-on (early, 13–120 days) and long-term (long, > 120 days) neoadjuvant aromatase inhibitor therapy with letrozole. ***p < 0.001; *p < 0.05. NA not available, Rec+ recurrence, Rec– recurrence free

The above findings suggest that therapy-induced dynamic changes in gene expression and pathways are common features of long-term treatment, rather than being specific to dormant or resistant phenotypes. This led us to perform comparative analysis of dormant and acquired resistant tumours at the long-term time point to identify any specific differences. Unpaired rank product analysis (FDR < 0.01) revealed a total of 419 genes (170 downregulated and 249 upregulated) to be differentially expressed between long-term treated dormant and resistant tumours (Additional file 2: Table S6; Fig. 4a). These genes were significantly enriched in 27 pathways (*p* < 0.05), including several epigenetics-related pathways, including “DNA methylation”, “PRC2 methylates histones and DNA”, “histone acetyl transferases (HATs) acetylate histones”, and “epigenetic regulation of gene expression”, as well as senescence and cell cycle (Additional file 2: Table S7; Fig. 4b). Examination of the expression of these genes alone demonstrated that they could partially separate dormant from the majority of resistant tumours (Fig. 4c). Single-sample gene set enrichment analysis (ssGSEA) [33] was performed to quantitatively score the activity of differentially expressed genes in every sample. The score of differentially upregulated genes between long-term treated dormant and resistant tumours was significantly higher in acquired resistant compared with dormant tumours under early treatment (*p* < 0.05) as well as long-term treatment (*p* < 0.001) (Fig. 4d).

**Fig. 5:**
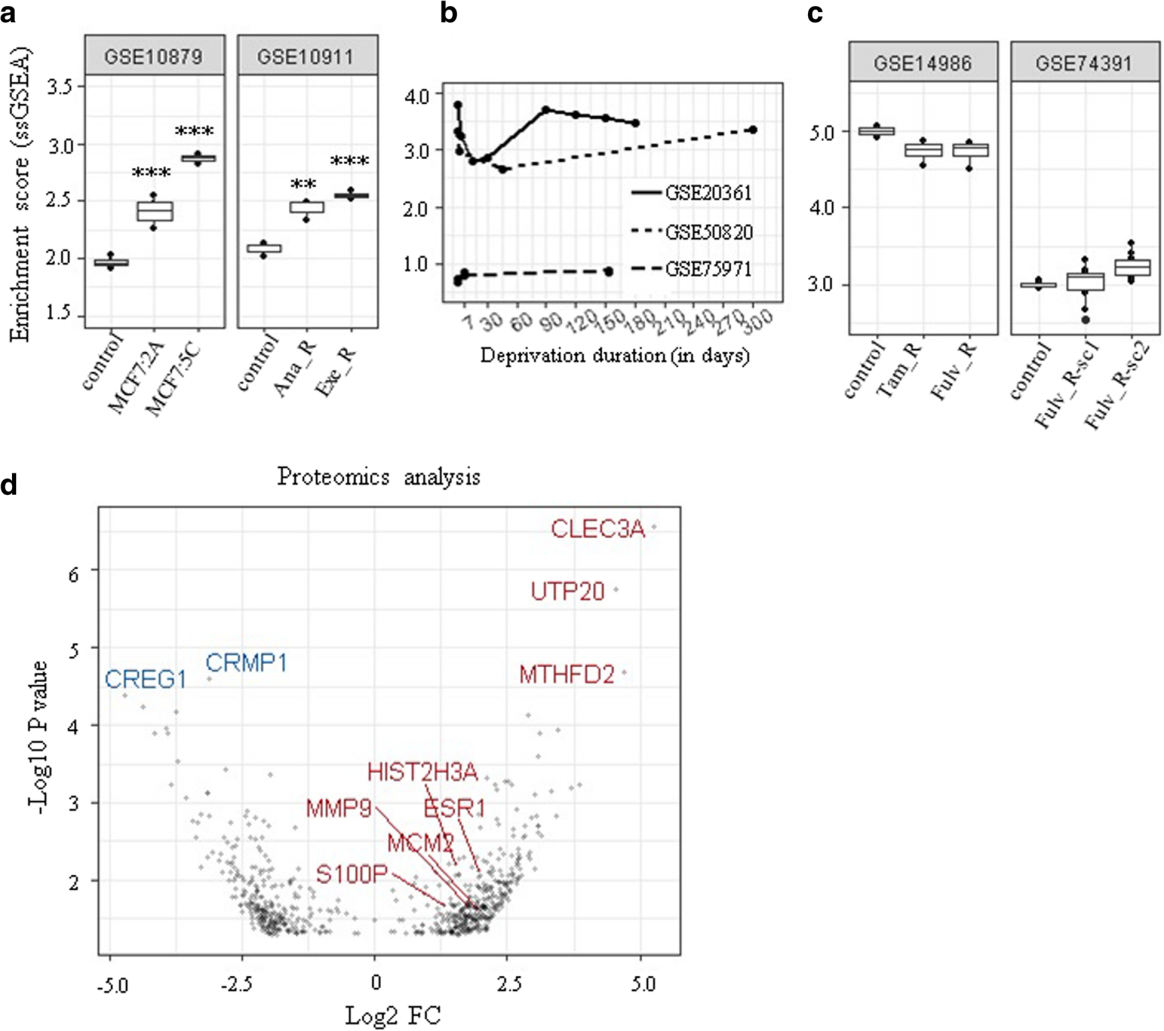
Validation of results using in-vitro gene expression data from resistant cell lines and proteomics analysis. **a** Normalised enrichment scores of differently upregulated genes (a total of 249) between long-term treated dormant and resistant tumours calculated using single sample gene set enrichment analysis (ssGSEA) in aromatase inhibitor-resistant cells. Scores were significantly higher (**p < 0.01, ***p < 0.001) in two aromatase inhibitor-resistant cell lines, MCF7:2A and MCF7:5C, which were clonally derived from MCF7 breast cancer cells following long-term oestrogen deprivation (LTED) compared with control/sensitive MCF7 cells (*n* = 4). Anastrozole-resistant (Ana_R) and exemestane-resistant (Exe_R) MCF7aro cells had significantly higher scores compared with control (*n* = 3). **b** Dynamic changes in enrichment scores of LTED MCF7 cells in three different datasets. **c** Scores in tamoxifen-resistant (Tam_R) and fulvestrant-resistant (Fulv_R) and drug-sensitive (control) MCF7 cells (*n* = 4, *n* = 10). **d** Volcano plot showing differentially expressed proteins between long-term treated dormant and resistant tumours (*p* < 0.05). Some overlapping features between transcriptomics and proteomics analysis and the most upregulated and downregulated proteins are highlighted in red and blue, respectively. FC fold-change, sc subclone

Our results prompted us to examine whether the changes we observed in clinical samples were similarly changed in experimental models of resistant breast cancer cells. Oestrogen receptor-positive MCF7 cells stably transfected with the aromatase gene (MCF7aro cells) and long-term oestrogen-deprived (LTED) breast cancer cells have been widely used to understand mechanisms of aromatase inhibitor resistance in vitro. Examining two publicly available gene expression datasets (GSE10879 and GSE10911) demonstrated that genes differentially expressed (upregulated) between acquired resistant and dormant tumours (a total of 249) were significantly enriched in aromatase inhibitor-resistant cells compared with sensitive/control cells (Fig. 5a). A total of 211 and 174 out of 249 genes were present in GSE10879 and GSE10911, respectively. In two out of three in-vitro studies with dynamic gene expression data from LTED MCF7 cells, an initial decrease in ssGSEA scores mimicking the dormancy/responsive state was followed by a later increase representing acquired resistance (Fig. 5b), further validating our results and emphasizing the utility of these in-vitro models. Interestingly, no significant difference was observed in tamoxifen- and fulvestrant-resistant MCF7 cells compared with drug-sensitive control cells (Fig. 5c) suggesting the specificity of the results to resistance to aromatase inhibitor therapy.

In addition, proteomic analysis of a subset of samples was performed which revealed differential expression in 656 proteins (279 downregulated, 377 upregulated) between long-term treated dormant and resistant tumours (rank product; *p* < 0.05; *n* = 10; Additional file 2: Table S8; Fig. 5d). A total of 36 features including S100P and HIST2H3A (H3.2) overlapped between proteomics and transcriptomics, validating the results with a different approach.

Furthermore, differentially expressed genes were uploaded to Enricher (ENCODE Histone modification 2015 dataset) [34] to determine histone modification enrichment. Two H3 lysine methylation modifications (H3K27me3 and H3K4me1) were enriched significantly (adjusted *p* = 0.0003 and *p* = 0.004, respectively) whereas no enrichment for histone acetylation was determined.

**Fig. 6:**
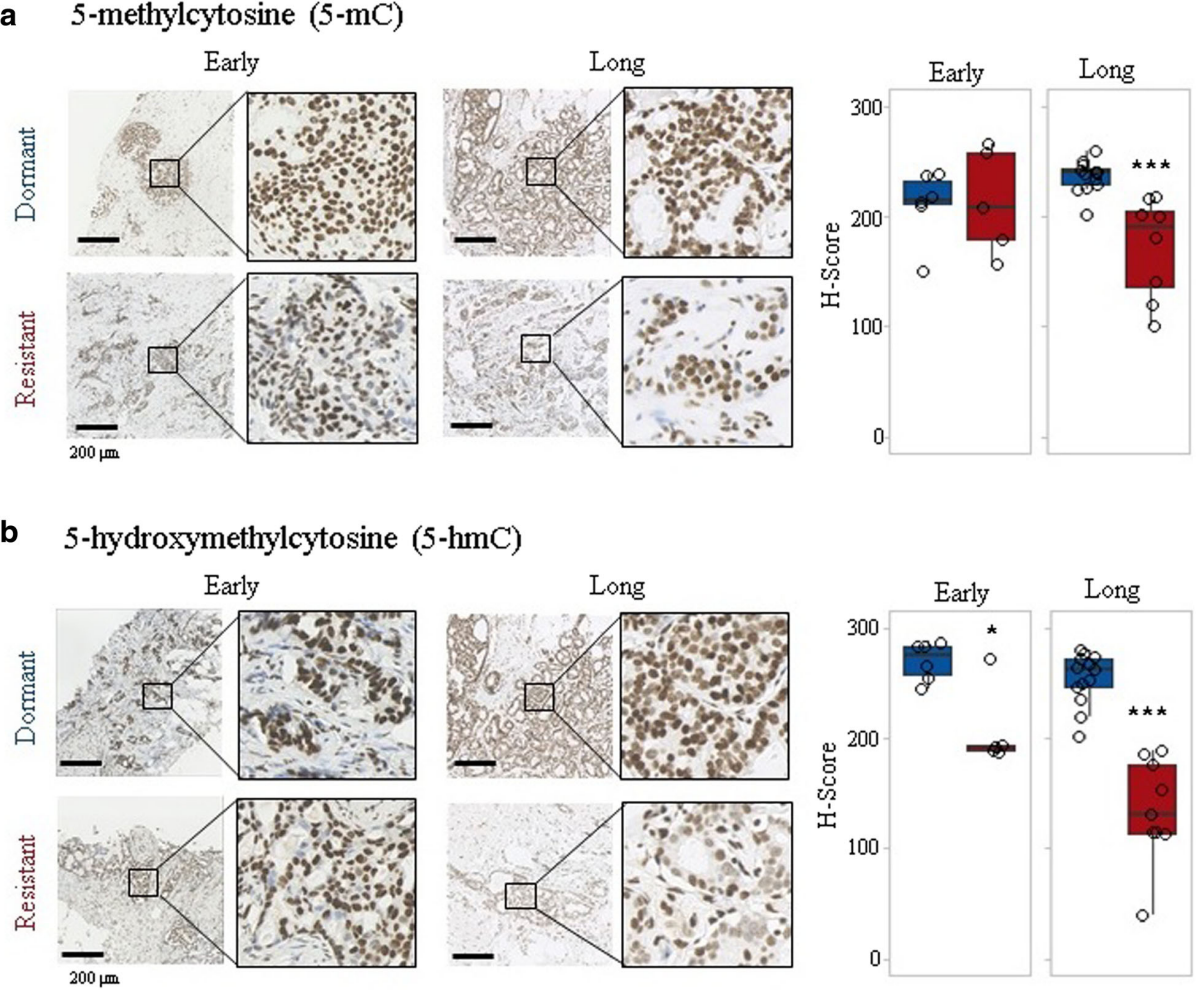
Immunohistochemical evaluation of methylation markers. **a** 5-methylcytosine (5-mC) and **b** 5-hydroxymethylcytosine (5-hmC) levels were determined in FFPE sections from letrozole-treated samples. Representative images in dormant and resistant tumours are shown. Boxplots show distributions of semi-quantitative intensity scores of 5-mC (*p < 0.05, ***p < 0.001; *n* = 5–12) and 5-hmC (***p < 0.001; *n* = 5–13) levels in dormant and resistant tumours. Early-on (early, 13–120 days) and long-term (long, > 120 days) neoadjuvant aromatase inhibitor therapy with letrozole

To further validate the gene expression results, immunohistochemical evaluation of FFPE sections revealed significantly lower global 5-mC and 5-hmC levels in resistant tumours compared with dormant tumours under extended treatment (Fig. 6a, b). Significantly lower 5-hmC levels in acquired resistant compared with dormant tumours were also observed at early treatment (Fig. 6b), suggesting hypomethylation may be predictive of emergence from dormancy.

## Discussion

Understanding the mechanisms underlying the maintenance of and escape from dormancy have great importance considering that most cancer-related deaths are caused by metastasis rather than the primary tumour. In this study, we describe the first sequential patient-matched clinical dataset of extended oestrogen deprivation therapy in breast cancer. The results highlight the difficulty of distinguishing dormant and resistant tumours, with dynamic molecular changes of treatment being highly similar between the groups. However, comparative analysis revealed a set of genes significantly upregulated in resistant tumours compared with dormant tumours within the first months of letrozole treatment suggesting a predictive role for changes in DNA methylation.

Failure to reduce proliferation after 2 weeks of oestrogen deprivation therapy [16, 35] may well identify patients that are innately resistant; however, acquired resistance remains a greater challenge in terms of identifying biomarkers and appropriate alternative or combination therapies [36]. Many of the transcriptomic changes identified in long-term treated dormant tumours are shared by some, but not all, resistant tumours, providing further evidence of resistance heterogeneity [37] where dormant tumours share similar molecular changes, but there are likely to be a variety of escape mechanisms that lead to acquired resistance.

In the present study, paired differential expression analysis demonstrated that dormant tumours continue to change under long-term treatment. Some of the identified dormancy-related pathways such as cell cycle arrest and senescence have established roles in metastasis dormancy [38], further supporting the relevance of our clinical model, with the senescence-associated secretory phenotype (SASP) recently suggested to regulate breast cancer dormancy and relapse [39]. As in short-term responsive tumours [16], ECM organization and degradation were significantly upregulated in dormant tumours. ECM remodelling and its degradation by matrix metalloproteases (MMP) have previously been suggested to regulate the switch between dormancy and metastatic growth [40]. Despite histological confirmation that each tumour sample contained at least 60% tumour, we acknowledge that the results presented are of intact whole tissue and potentially limited by minor variations in tumour cellularity or the proportion of stoma which could affect gene expression.

The most transcriptionally upregulated gene in resistant tumours *S100P*, previously shown to be an inducer of breast cancer metastasis correlated with decreased survival [41]. S100P, a small calcium-binding protein mediating Ca^2+^-dependent signalling pathways, has distinct functions in normal tissue and cancer, including human embryonic development and breast cancer initiation [42]. Recently, *S100P* hypomethylation in blood was demonstrated to be inversely correlated with tissue *S100P* expression and significantly associated with breast cancer, implicating *S100P* as a potential diagnostic marker [43]. High plasma *S100P* levels have also been correlated with poor prognosis in metastatic breast cancer patients, with levels decreasing following treatment, suggesting a role of S100P in dynamic monitoring of response [44]. In the present study, S100P gene expression and protein levels were significantly higher in resistant tumours after long-term treatment, as well as being differentially expressed before treatment, supporting its potential role as a therapeutic target [45] and a predictive marker.

Comparative analysis of dormant and resistant samples after extended treatment revealed enrichment for a set of genes with a role in DNA methylation and histone acetylation/deacetylation. Epigenetic alterations are recognized to occur in breast cancer. DNA methyltransferase (DNMT) and histone deacetylase (HDAC) inhibitors have been shown to exert encouraging effects on the disease [46]. Recently, the potential role of epigenetic changes in regulating dormancy and reactivation state has been suggested to explain the reversible (on/off) nature of dormancy [47].

Breast cancer “CpG island methylator phenotype” (CIMP), as revealed by genome-wide methylation analysis of metastatic breast cancers where a large number of genes are hypermethylated, has been suggested to be informative for metastatic potential [48]. A significant correlation between pre-treatment global DNA methylation with neoadjuvant chemotherapy response in rectal cancer has been reported [49]. Although DNA hypomethylation was the first epigenetic alteration identified in cancer, its molecular process and effects are not yet well understood [50]. In addition, 5-hmC levels were shown to correlate with differentiation status, with higher levels when more differentiated [29]. In addition, alterations in DNA methylation in LTED MCF7 cells have been previously reported [51]. Our results provide evidence for the loss of a global DNA methylation process in resistant tumours and strengthen the case to use these models for further study. The global decrease in 5-mC may account for the observed reduction in 5-hmC levels since 5-mC is converted to 5-hmC. On the other hand, at the early-on time point, 5-hmC levels were significantly reduced with no significant change in 5-mC levels, suggesting an independent role of the 5-hmC mark. Hypomethylated cancer cells have been suggested to be selected to form tumours with increased malignancy [50]. We suggest that hypomethylation in resistant tumours may reflect a de-differentiation process inducing stem cell-like cell formation. Determining the time point at which that hypomethylation starts, which would allow intervention before it starts to prevent resistance to therapy, needs further investigation.

The main genes significantly enriched for epigenetics-associated pathways in the present study are core histone (H3, H4, H2B) genes. Well-known epigenetics-associated genes such as DNMT were not differentially expressed in the present study. Therefore, it might be suggested that observed changes in histone gene levels may simply reflect the high proliferation rate in resistant tumours since transcription of these histone genes are replication-dependent and their mRNA levels increase during DNA replication [52]. However, deregulation of histone H2A and H2B was associated with anthracycline resistance in breast cancer cells and reversed by HDAC small molecule inhibitors [53]. Furthermore, upregulation of replication-dependent core histone proteins has been suggested to be a selective indicator of ER-mediated MCF7 cell proliferation regardless of the proliferation rate [54]. Also, observed global loss of DNA methylation in resistant tumours suggests dynamic regulation of gene transcription under letrozole therapy. Therefore, histone upregulation and alterations in epigenetic pathways observed in our study may play a role in resistance to endocrine deprivation therapy, rather than simply mirroring the degree of proliferation.

Our results indicate alterations both in DNA methylation and histone modifications. HDAC inhibitors, which have been shown to regulate DNA methylation [55], may be successful clinically as second-line drugs alone or in combination following oestrogen deprivation therapy failure as there is growing evidence for their tumour selective action [56, 57]. A time-dependent role for HDACs in leukaemia has been shown [58] and may also be critical in determining when to start HDAC inhibition therapy to successfully treat tumours resistant to oestrogen deprivation therapy. Whether or not the epigenetic alterations are triggers of re-awakening and if the timely use of epigenetic drugs can prevent acquired resistance warrants further investigation.

## Conclusions

We have performed the first study of sequential tumour samples from breast cancer patients receiving extended neoadjuvant oestrogen deprivation therapy as a clinical model of dormancy and acquired resistance. Our analysis suggests that molecular differences between dormant and resistant tumours are initially subtle, becoming more obvious only after extended treatment. This study emphasizes that alterations in DNA methylation in the first months of treatment may predict which patients will eventually develop acquired resistance.

## Additional files

Additional file 1: **Figure S1.** Consort diagram showing the cohort and sample sizes. Patient and sample sizes in each group are shown with inclusion and exclusion criteria. (JPG 80 kb)

Additional file 2: **Table S1.** Table indicating with which public dataset each sample is associated. **Table S2.** Differentially expressed genes between long-term treated and pre-treatment dormant tumours. **Table S3.** Enriched pathways for differentially expressed genes between long-term treated and pre-treatment dormant tumours. **Table S4.** Differentially expressed genes between long-term treated and pre-treatment acquired resistant tumours. **Table S5.** Enriched pathways for differentially expressed genes between long-term treated and pre-treatment acquired resistant tumours. **Table S6.** Differentially expressed genes between long-term treated dormant and resistant tumours. **Table S7.** Enriched pathways for differentially expressed genes between long-term treated dormant and resistant tumours. **Table S8.** Differentially expressed proteins between long-term treated dormant and resistant tumours. (XLSX 286 kb)

## Abbreviations

5-hmC: 5-Hydroxymethylcytosine
5-mC: 5-Methylcytosine
CIMP: CpG island methylator phenotype
DAB: 3,3′-Diaminobenzidine
DNMT: DNA methyltransferase
ECM: Extracellular matrix
ER: Oestrogen receptor
FASP: Filter Aided Sample Preparation
FDR: False discovery rate
FFPE: Formalin-fixed paraffin-embedded
HAT: Histone acetyl transferase
HDAC: Histone deacetylase
HRP: Horseradish peroxidase
LFQ: Label-free Quantitation
LTED: Long-term oestrogen deprived
MDS: Multidimensional scaling
MMP: Matrix metalloproteases
SASP: Senescence-associated secretory phenotype
ssGSEA: Single-sample gene set enrichment analysis
