# Supplementary figures and images for: Molecular changes during extended neoadjuvant letrozole treatment of breast cancer: distinguishing acquired resistance from dormant tumours
Source: Breast Cancer Res. 2019 Jan 7;21:2. doi: 10.1186/s13058-018-1089-5 (PMC6323855; doi:10.1186/s13058-018-1089-5)

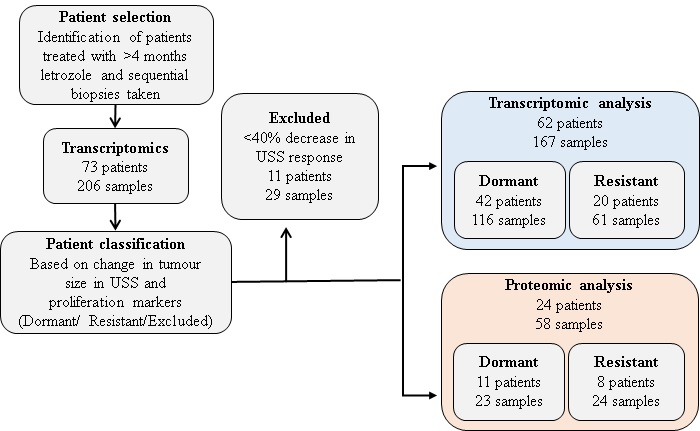

Supplement: Supplementary file 1 — Figure S1. Consort diagram showing the cohort and sample sizes. Patient and sample sizes in each group are shown with inclusion and exclusion criteria. (JPG 80 kb) [file 13058_2018_1089_MOESM1_ESM.jpg]
